# Antioxidant Activity of Thyme Waste Extract in O/W Emulsions

**DOI:** 10.3390/antiox8080243

**Published:** 2019-07-25

**Authors:** Soukaïna El-Guendouz, Smail Aazza, Susana Anahi Dandlen, Nessrine Majdoub, Badiaa Lyoussi, Sara Raposo, Maria Dulce Antunes, Vera Gomes, Maria Graça Miguel

**Affiliations:** 1 Laboratory of Physiology-Pharmacology-Environmental Health, Faculty of Sciences Dhar El Mehraz, BP 1796 Atlas, University Sidi Mohamed Ben Abdallah, Fez 30 000, Morocco; 2 Departamento de Química e Farmácia, MeditBio, Faculdade de Ciências e Tecnologia, Universidade do Algarve, Campus de Gambelas, 8005-139 Faro, Portugal; 3 Laboratory of Phytochemistry, National Agency of Medicinal and Aromatic Plants (ANPMA). BP. 159, Principal, Taounate 34000, Morocco; 4 Centre for Marine and Environmental Research (CIMA), Faculdade de Ciências e Tecnologia, Universidade do Algarve, Campus de Gambelas, 8005-139 Faro, Portugal; 5 MeditBio/CEOT, Faculdade de Ciências e Tecnologia, Universidade do Algarve, Campus de Gambelas, 8005-139 Faro, Portugal; 6 Centre of Marine Sciences (CCMAR), Universidade do Algarve, Campus de Gambelas, 8005-139 Faro, Portugal

**Keywords:** recycling aromatic waste, almond oil, wheaty germ oil, oxidative stability, response surface methodology

## Abstract

*Thymus vulgaris* (thyme) is an aromatic plant and its essential oil has been applied as antimicrobial and antioxidant due to the presence of phenolic compounds. However, after steam distillation, the deodorized plant material is rejected, despite the possible presence of bioactive compounds. Ethanolic thyme waste extract revealed the presence of benzoic acid, 4-hydroxybenzoic acid, ferulic acid, caffeic acid, and sinapic acid. This waste thyme extract had the capacity for preventing the formation of primary and secondary lipid oxidation products in emulsions O/W (oil in water), constituted by diverse proportions of wheat and almond oils, without interfering with the viscosity parameters, for 10 weeks, at 37 °C. The increasing proportion of almond oil (≥50%) in the emulsion increases its resistance to oxidation, which is improved with the presence of an optimal concentration of tested thyme waste extract (0.02% and 0.04%). The waste thyme extract can, therefore, be used as an antioxidant either in food or pharmaceutical emulsions O/W, replacing the synthetic antioxidants.

## 1. Introduction

In spite of emulsions O/W being largely used in food, pharmaceutical, cosmetic industries, and biomedical sciences [1], they are thermodynamically unstable, tending to breakdown through coalescence and aggregation of the dispersed oil phase, over time [2]. Oxidation of the lipid phase of the emulsion O/W may occur, generating oxidative sub-products which interfere in the emulsion stability and unpleasant odorant compounds that impair the quality of the final product. The formation of free radicals can be avoided or decreased by adding antioxidants [3]. However, their use cannot be random and should be chosen taking into account their physicochemical properties, so that they preferentially remain at the interface O/W [3].

Tocopherols, butylhydroxy toluene (BHT) or butylhydroxy anisole (BHA), gallates, and plant phenolics (quercetin or rosmarinic acid) have been used as antioxidants in emulsions O/W [4,5]. Thyme (*Thymus vulgaris* L.) is a source of a variety of natural bioactive materials, it contains many flavonoids, like lutein, apigenin, naringenin, luteolin, and thymonin [6], beyond thymol and carvacrol in the volatile part of the plant [7]. These two phenolic compounds have been considered as good antioxidants [7]. The large production of essential oils originates waste that is usually poorly exploited. Such can be reversed and the deodorized plant that remains after the distillation may still contain bioactive compounds with antioxidant activity that must be valued [8].

In the Algarve (Portugal), there is an essential oils’ producer that struggles against the waste that remains after steam distillation. Despite the extraction of the volatile fraction, the remaining solid material still contains bioactive compounds. Some studies have shown that post-distillation waste material from thyme (*Thymus vulgaris*) still possess antioxidant and antimicrobial activities [9,10]. In spring and summer, there is production of essential oils obtained from the flowering aerial parts from diverse aromatic plants collected in organic agriculture farmers of Algarve, such as *Thymus vulgaris*. The goal of the present work was to evaluate the antioxidant capacity of an extract of deodorized *Thymus vulgaris* (thyme) by-product, obtained by a Portuguese producer of essential oils, in simple emulsions O/W in which the lipid phase was constituted by almond or wheat germ oils (natural products). The viscosity of the emulsion behavior type was also studied. 

## 2. Material and Methods

### 2.1. Materials

Thiobarbituric acid (TCA), ferrous chloride (FeCl_2_), ammonium thiocyanate (NH_4_SCN), ferulic acid, DNP (2,4-dinitrophenylhydrazine), sulphuric acid (H_2_SO_4_), eryodictiol, quercetin, potassium hydroxide (KOH), *N*-methyl-*N*-(trimethylsilyl) trifluoroacetamide, NH_4_I, 2-mercaptoethanol, and buthylated hydroxyanisole (BHA) were purchased from Fluka Biochemika, Sigma-Aldrich, Steinheim, Germany. 2,2-diphenyl-1-picrylhydrazyl (DPPH) was from Riedel-de Haën, Sigma-Aldrich, Seelze, Germany. Folin–Ciocalteu’s phenol reagent and AlCl_3_ were purchased from Panreac Quimica, Montcada i Reixac, Barcelona, Spain. Na_2_CO_3_ was purchased from Riedel de Haen (Seelze, Riedel-de-Haën Laboratory chemicals, Germany). Almond oil and wheat germ oil were purchased from LBCHEM Labospirit, Lda Portugal. Tween 80, Span 80, Phenonip and xanthan gum were purchased from Guinama, Valencia, S.L.U Spain. Trichloroacetic acid was purchased from VWR, Leuven, Belgium. 1-Butanol, methanol, and HCl were from Fisher Scientific UK Ltd., Loughborough, UK. 

### 2.2. Plant Extract

Solid material resulting from steam distillation of the flowering aerial parts of *Thymus vulgaris* L. from an organic agriculture in the Algarve region, Portugal, was a gift from a local producer of essential oils. That material was dried over 7 days at room temperature and then ground. Afterward, thyme was extracted with ethanol using Sohxhlet extraction method where 20 cycles were done before being concentrated using a rotavapor and kept until analysis at −4 °C in dark.

### 2.3. Chemical Composition of Thyme Waste Extract

Sample preparation: 100 µL of ethanolic sample (thyme waste extract) was evaporated with nitrogen stream and derivatized by the addition of 50 µL of the derivatization reagent. Afterwards, the sample was heated at 60 °C for 30 min. The derivatization agent was prepared as follows: an intermediate stock solution (Sol A) was prepared with 0.25 mL of *N*-methyl-*N*-(trimethylsilyl) trifluoroacetamide (MSTFA) mixed with 10 mg NH_4_I and 15 μL of 2-mercaptoethanol. Then, the Sol A was mixed with pure MSTFA (1/9). Compounds’ identification was performed using retention times and spectra from a lab-made library, created with commercial standards also previously submitted to the same derivatization procedure.

The gas chromatography/mass spectrometry (GC) unit was a Bruker Scion 456-GC TQ with Phenomenex ZB5-MS capillary column (30 m × 0.25 mm ID, 0.25 μm df) at the following conditions: Sample volume: 1 μL, temperature. of injector: 280 °C; flow of He 1.0 mL/min; Program temperature: 80 °C, hold: 1 min, 220 °C and increase 10 °C/min; 310 °C and increase 20 °C/final mine hold 7 min, in a total run time of 26.50 min. Source: 220 °C; temperature of transfer line: 260 °C; mass range: 50–350 (*m*/*z*). The software used was Bruker Daltonics MS Worksation version 8.2.1. and MS Search 2.0. (National Institute of Standards and Technology, Gaithersburg, MD, USA).

### 2.4. Total Phenolics, Flavones, Flavonols, Flavanones, and Dihydroflavonols Content Determination in Thyme Extract

The total polyphenols content in thyme waste extract was determined using the El-Guendouz et al. method [11] with slight modification: 250 μL of extracts or standard (gallic acid at different concentrations) were mixed with 1 mL of 7.5% Na_2_CO_3_ and 1.25 mL of Folin-Ciocalteu reagent (0.2 N). The mixture was well vortexed and after 2 h of incubation at room temperature, the absorbance was measured at 760 nm. The amount of flavones and flavonols was determined according to the El-Guendouz et al. method [11]: 500 μL of thyme waste extract or standard (quercetin at different concentrations) were added to a volume of 500 μL of AlCl_3_ (2%) and after one hour at room temperature the absorbance was measured at 420 nm. For the total amount of flavanones and dihydroflavonols compounds, the use of 2,4-dinitrophenylhydrazine (DNP) was determined, and the absorbance was measured at 486 nm as reported by El-Guendouz et al. [12] with some modifications. Briefly, 150 µL of sample or standard (eriodyctiol at different concentrations) and 100 μL of acidic DNP methanolic solution 1% were heated at 50 °C for 50 min in a water bath. After cooling at room temperature, the mixture was diluted to 10 mL with methanolic solution of KOH 10% (*w*/*v*). One mL of the resulting solution was added to 10 mL methanol and diluted to 50 mL with methanol. 

Each experiment was carried out in triplicate. A calibration curve was used and the results were expressed as mg/g of gallic acid equivalents (GAE), mg/g of quercetin equivalents (QE) and mg/g of eriodyctiol equivalents (EE) for the quantification of phenols, flavones and flavonols, and dihydroflavonols, respectively.

### 2.5. Antioxidant Activity Determination of Thyme Waste Extract

The antioxidant capacity of thyme was measured through DPPH assay according to the methods previously described [13]. 

### 2.6. Preparation and Storage of O/W Emulsion

Oil-in-water (O/W) emulsions were prepared as described in our previous work [14], but replacing propolis by thyme waste extract, where three different concentrations were used (0.01, 0.02 and 0.04%), and five different formulations of oils’ phases content (A: 100% of wheat germ oil/0% almond oil; B: 75% of wheat germ oil/25% almond oil; C: 50% of wheat germ oil/50% almond oil; D: 25% of wheat germ oil/75% almond oil and E: 0% of wheat germ oil/100% almond oil) for each concentration of thyme waste extract. BHA was used as positive control. 

### 2.7. Viscosity Studies

Viscosity studies of the different samples were determined using a Brookfield programmable rotational viscometer LVDV-II+Pro (Brookfield Engineering Laboratories Inc., Middleboro, MA, USA) equipped with the Rheocalc 32 (Brookfield Engineering Laboratories Inc., Middleboro, MA, USA) (version 2.4.) software as previously reported [14]. The shear data were analyzed according the power-law equation (τ = K. γ^n^) to obtain the consistency index (*K*) and the flow behavior index (*n*) for the different emulsions.

### 2.8. Peroxide Content Determination

Peroxide content determination was done at 0, 2, 4, 6, 8, and 10 weeks as described by El-Guendouz et al. [14] by measuring lipid hydroperoxide at the absorbance 510 nm. Three independent determinations for each sample were carried out.

### 2.9. Lipid Oxidation Products in Formulated Emulsions

Thiobarbituric acid-reactive substances (TBARS) were determined on the emulsions after 2, 4, 6, 8, and 10 weeks of incubation at 37 °C, according to El-Guendouz et al. [14] method. Three independent determinations for each sample were carried out. 

### 2.10. Experimental Design and Statistical Analysis

Viscosity analyses were performed on duplicated samples. Statistical analysis was conducted with a SigmaPlot 12 software (Systat Software, version 12 for Windows) (Systat, San Jose, CA, USA), implementing the one-way ANOVA method. Significant differences among means (*p* < 0.05) were determined by Student-Newman-Keuls test.

Central composite design (CCD) of the central response surface methodology (RSM) was used to study the effect of four independent variables in different combinations, X1 (BHA concentration %), X2 (thyme waste extract concentration %), X3 (almond oil/wheat germ oil ratio %), and X4 (storage time in weeks) on lipid peroxidation (dependent variable), using STATISTICA (Stat Soft. InC., Tulsa, OK, USA), version 10, Trial Version. All experiments were conducted in triplicate, data were analyzed by the analysis of variance (ANOVA), and *p*-value lower then 0.05 was considered significant in surface response analysis. A total of 210 experiments were used to calculate the coefficient of the second-order polynomial regression of the four variables, and the model suitability was tested using the ANOVA test. A second-order polynomial equation was used to express the lipid peroxidation and secondary lipid oxidation (DO) (*Y*) as a function of the independent variables as

$$y=\beta_{0}+\sum_{i=1}^{4}\beta_{i}x_{i}+\sum_{i=1}^{4}\beta_{ii}x_{i}^{2}+\sum_{i<j}^{4}\beta_{ij}x_{i}x_{j}$$

where *y* represents the response variables; *β*_0_ is a constant; *β_i_*, *β_ii_*, and *β_ij_* are the linear, quadratic, and cross-product coefficients, respectively. *x_i_* and *x_j_* are the levels of the independent variables. Two-dimensional surface response contour plots were generated by varying two variables within the experimental range and holding the other two constants at the central point.

The regression model for the formation of peroxides in the formulated emulsions is presented in the following equation:

*Peroxide content* = 1.214 − 3.009 TWE + 24.5 TWE^2^ − 5.9 × 10^−4^ AO +1.86 × 10^−8^ × AO^2^ + 1.186 × 10^−2^ × BHA × AO + 0.10 × TWE × AO + 0.015 × W × TE + 4.41 × 10^−5^ × W × AO

where TWE: Thyme waste extract (TWE); AO: Almond oil %; W: storage time (weeks); BHA: Butylated hydroxyanisole.

The regression model for secondary lipid oxidation products’ content in the formulated emulsions is presented in the following equation:

*Absorbance* (TBARS) = 1.25 − 42.26 TWE + 628.59 TWE^2^ − 0.0069 AO − 3.38 10^−4^ AO^2^ + 0.169 × BHA × TWE + 0.160 TWE × AO − 1.086 × W × TWE − 7.08 × 10^−4^ × W × AO.

where TWE: Thyme waste extract; AO: Almond oil %; W: storage time (weeks); BHA: Butylated hydroxyanisole.

## 3. Results and Discussion

### 3.1. GC–MS Analysis of Thyme Waste Extract

The chemical composition of thyme waste extract, determined by GC/MS, is displayed in Table 1. Nine components representing 96.19% (i.e., relative %, rel. % of the total detected constituents) were identified in this extract. The main compound in the examined thyme waste extract was benzoic acid, which presents 40.88% (relative percentage). The amounts of 4-hydroxybenzoic acid, *trans*-cinnamic acid and *p*-coumaric acid found were 17.18%, 11.89%, and 10.34 rel%, respectively. Other identified phenolic acids were ferulic, caffeic, and sinapic acids. Quercetin was also detected in this extract. Similar studies in Romania identified caffeic, chlorogenic, *p*-coumaric, and ferulic acids, luteolin, and apigenin as major compounds in the *T. vulgaris* extract [15]. In the deodorized leaves of thyme. rutin and apigenin were found as main phenols, along with quercetin, chlorogenic, ferulic, and gallic acid [9].

### 3.2. Polyphenol Contents and Antioxidant Activity

Table 1 depicts the total phenolics and flavonoids contents of thyme extract. The sample exhibited 97.50 ± 3.82 mg GAE/g (total phenols), 1.76 ± 0.24 mg EE/g (flavanones and dihydroflavonols), and 0.13±0.01 mg QE/g (flavones and flavonols). The results agree with the literature, which reports the phenolics content of thyme to be relatively high in different extracts [16,17]. 

Several studies have indicated that phenolic contents are strongly related to antioxidant activities. According to Gülçin et al. [18], a high level of phenolic compounds indicates the elevated antioxidant capacity of thyme. Antioxidant activity was tested through DPPH test, and the IC_50_ was 93 μg/mL, similar to that of BHT (89 μg/mL), used as the control. Lower IC_50_ values (better activity) were found (13.4 µg/mL and 12.1 µg/mL) by other authors regarding DPPH scavenging activity of thyme extract [16]. Abdul et al. [19] reported IC_50_ values ranging from 48.1 µg/mL to 59.3 µg/mL, these values are higher than those found by Köksal et al. [16] but still lower than those found in our extract. The higher IC_50_ values (lower activity) found in the present work cannot be compared to those reported by [16,19], since they studied the antioxidant activity of thyme extracts and in the present work, the antioxidant activity was determined in thyme waste extract in which bioactive compounds (thymol and/or carvacrol) were previously removed during the volatiles’ extraction, by steam distillation. The transformations undergone by some compounds during distillation cannot also be excluded. Gavarić et al. [9] found lower antioxidant activity in deodorized leaf extracts of thyme than the extracts without previous treatment. According to Gavarić et al. [9] who studied the antioxidant activity of pure compounds and compared them with those of waste extracts, concluded that the antioxidant activity of these samples could be attributed to the relative high amounts of rosmarinic acid and rutin.

As our extract still possessed antioxidant activity, it was assayed as antioxidant in the emulsions.

### 3.3. Viscosity of O/W Emulsion

Emulsions are thermodynamically unstable and they can be stabilized by surfactants or emulsifiers, or by thickening agents or stabilizers. Surfactants or emulsifiers and stabilizers can be combined and used for improving the stability of emulsions O/W [20]. In the present work, Tween 80 and Span 80, and xanthan gum were used as surfactants, and stabilizer, respectively.

**Figure 1:**
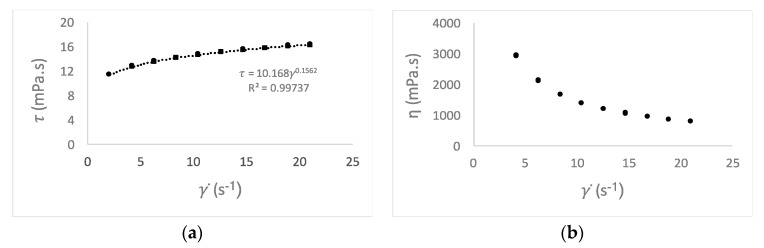
Flow behavior curve and power law model (^.....^) fitting (**a**) and relation between apparent viscosity and the shear rate (**b**), at 25 ± 1 °C with an up-down rate ramp.

**Figure 2:**
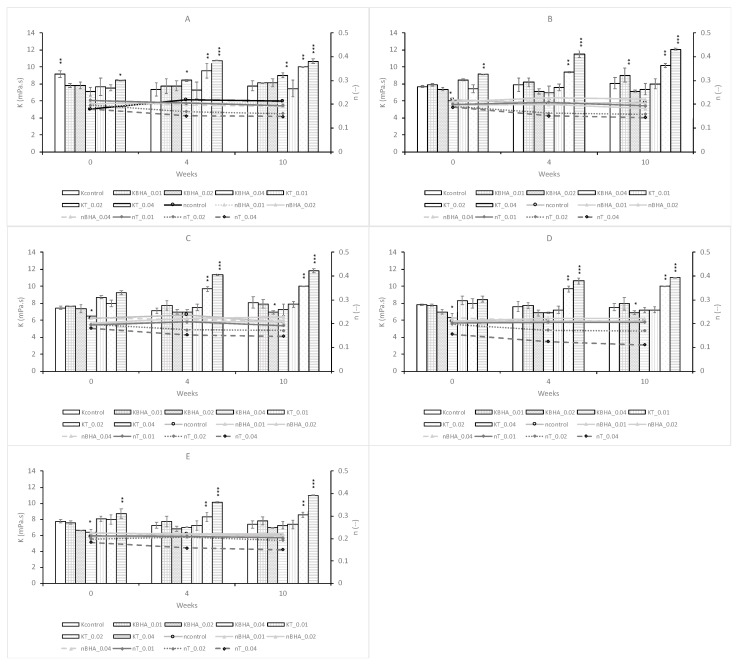
Effect of adding thyme extract and BHA, at different concentrations (0.01%, 0.02%, and 0.04%) on rheological characterization, with determination of consistency index (Kc, KBHA, KT) and fluid behavior index (nc, nBHA, nT), under storage conditions (37 °C for 10 weeks). Bars mean standard deviation (*n* = 4). Level of significance for the one-way ANOVA test: * *P* < 0.05; ** *P* < 0.01; *** *P* < 0.001. (**A**) 100% germ wheat oil and 0% almond oil. (**B**) 75% Germ wheat oil and 25% almond oil. (**C**) 50% Germ wheat oil and 50% almond oil. (**D**) 25% Germ wheat oil and 75% almond oil. (**E**) 0% Germ wheat oil and 100% almond oil.

The viscosity characterization was done for all emulsions O/W and was evaluated applying a shear rate from 2.10 to 21.00 s^−1^, with a 2.10 increment. Figure 1 presents the data to fit the power law model, relating the viscosity vs. shear rate to the determination of the consistency index (K), while the flow behavior index (*n*) is represented in Figure 2. In Figure 1a, the emulsions do not present thixotropic behavior, these mixtures present identical values of shear stress for the same shear rate value. The emulsions present a shear thinning behavior, by the representation of flow curves, where shear stress is fitting with shear rate (Figure 1a). In Figure 1b, the values of viscosity diminishes, when shear rate rises, demonstrating this shear thinning behavior.

The control and the different emulsions, with the waste thyme extract and BHA, have the values of consistency index (K) and the fluid behavior index (*n*) typical of shear thinning performance, with the index less than 1 (*n* < 0.22), which confirms the aforementioned results (Figure 2). 

As observed in Figure 2, the consistency index (K) for control remains constant over the 10 weeks of testing, presenting a slightly higher value in the formulation with 100% germ wheat oil, relative to BHA and waste thyme extract, with identical formulations (Figure 2A).

In general, for thyme formulations in all the storage weeks, the consistency index (K) is higher than the control and BHA formulations, having in week 0 a very similar value for different thyme waste extract concentrations. However, at week 4 and 10, the concentrations of 0.02 and 0.04 % presented a significant increase in the K, reaching the highest values for the concentration of 0.04%. This is reinforced with the viscosity values, since K index is a measure of the system consistency and is related to the emulsion viscosity. For the thyme waste extract (0.01%), the viscosity values are similar to those obtained with the other samples (results not shown). For the concentrations of 0.02% and 0.04% of thyme waste extract, after 10 weeks of storage, the higher viscosity values are 2136 ± 24 and 2451 ± 37 mPa s, respectively, against 1884 ± 57 mPa s for the control and 1722 ± 37 and 2046 ± 42 mPa s for BHA, respectively. These maximum values were obtained with 50% germ wheat oil and 50% almond oil. 

The composition of the oil percentage (wheat germ and almond oils), as the antioxidant added, influences the viscosity trend. As shown in Figure 2A–C, the initial viscosity increases with the decrease of wheat germ oil percentage, from 100% to 50%, for all the thyme waste extract concentrations. However, for higher concentrations (Figure 2D,E), the initial viscosity decreases with an increase in the almond oil percentage (Table S1). For the concentration of thyme extract 0.01%, a decrease of viscosity was observed with the 10 weeks of storage, as observed for the control. As reported in our previous work using propolis extract as an antioxidant [14], this decrease in viscosity could occur by the diffusion of water molecules from the internal to the external aqueous phase or for another reason, the bursting of multiple globules due to osmotic pressure [21]. Mahmood et al. [22] observed an identical decrease in viscosity in studies with multiple emulsions encapsulated with 5% green tea extract.

An opposite behavior was observed when the concentrations of thyme waste extracts were 0.02 and 0.04%: the viscosity increased with the storage time. The increase of the viscosity is noteworthy at the two concentrations of thyme sample, with the highest values being reached at 0.04% in the system constituted by equal parts of germ wheat and oil almond oil (2451 ± 37 mPa s). The storage conditions could contribute to this situation, the temperature of 37 °C, could enhance the evaporation of the aqueous phase of the emulsion. In addition, the hydro-alcoholic extracts may undergo higher evaporation, for instance, than aqueous extracts, causing increased viscosity and accentuating the shear thinning character, as visible by the curves of the thyme extract behavior index (Figure 1). A similar behavior was also observed for hydro-alcoholic extracts of propolis [14].

Likewise, after 10 weeks, comparing the different emulsions, based on the concentration of thyme waste extracts, it was found that the viscosity increases in all formulations tested. For the 0.01% concentration, the viscosity values vary between 1696 ± 27 and 1780 ± 39 mPa s, depending on the composition of the emulsion, while for 0.04%, the viscosity values vary between 2451 ± 37 and 2144 ± 49 mPa s, always after 10 weeks of storage.

Also, the flow behavior index (n) for thyme waste extract had a more pronounced shear thinning behavior (Figure 2). For the different formulations, thyme 0.02 and 0.04% concentrations present a decrease in the flow behavior index with the storage time, contrary to what is observed with the control or BHA. 

### 3.4. Peroxide Content Determination

Emulsion stability denotes the capacity to resist changes of physicochemical properties with the passage of time. It was reported that emulsion stability is strongly influenced by the concentration of the antioxidant agent and the types of oils used [23]. The optimization of all the main formulation factors is crucial for the successful preparation of stable emulsion products. Thus, in the present work, the effect of thyme waste extract on the oxidative properties of O/W emulsions formulated with two vegetable oils was examined. The emulsions were prepared using almond oil and wheat germ oil at different concentrations ranging from 0% to 100%, while thyme waste extract was chosen as a protector agent for its antioxidant properties, at concentrations ranging from 0.01% to 0.04%. 

**Figure 3:**
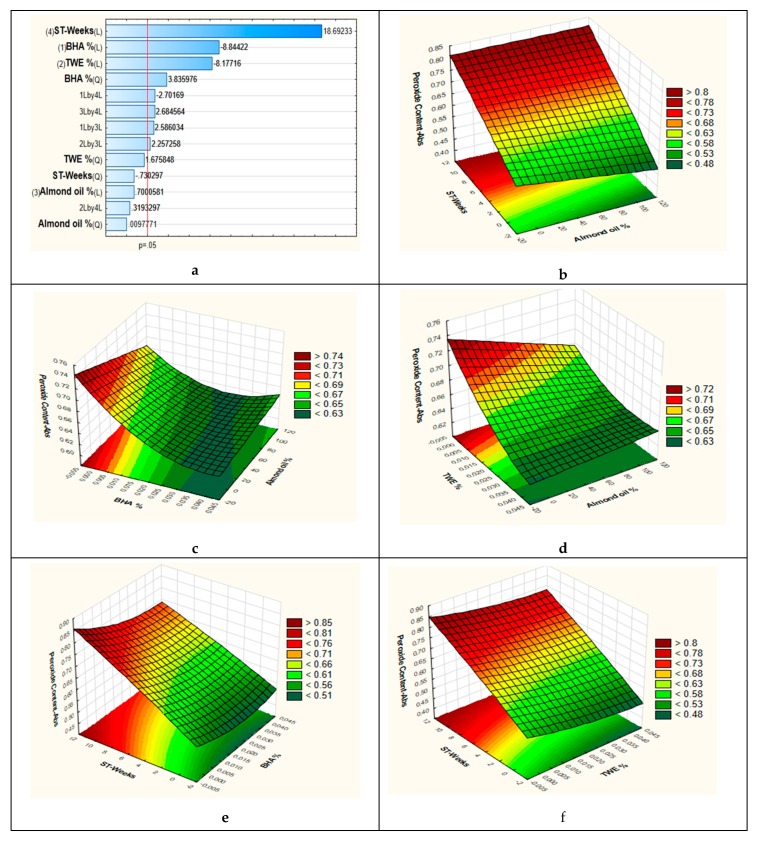
(**a**): Pareto chart showing the effects of the significant model coefficients. (**b**): Response surface plot showing the combined effect of storage time and emulsion almond oil %. (**c**): Response surface plot showing the combined effect of BHA concentration and emulsion almond oil %. (**d**): Response surface plot showing the combined effect of thyme extract concentration and emulsion almond oil %. (**e**): Response surface plot showing the combined effect of BHA concentration and storage time. (**f**): Response surface plot showing the combined effect of thyme extract concentration and storage time. TWE: Thyme waste extract; ST: storage time.

The results (Figure 3a) indicate that storage time, BHA concentration and thyme waste extract concentration in the emulsions had highly significant effects (*p* = 0.000000), and exerted great influence on the peroxide formation, whereas almond oil (L) and (Q) percentage in the emulsion did not produce a significant effect (*p* > 0.05). The cross-terms were statistically significant, except for 2 L by 4 L.

Three-dimensional surface plots were drawn to determine the interactive effect of the process variables on the formation of peroxides. The response surface plot of peroxide content in emulsions under different combinations is shown in Figure 3b–f. This set of dynamic graphs allowed us to evaluate the experimental interaction of different parameters on the formation of peroxides. Levels of peroxides in emulsions were found to increase highly with the storage time and slightly with the decrease of almond oil percentage (Figure 3b). The 3D plots for the combined effects of BHA concentration and almond oil percentage in the emulsion are given in Figure 3c. BHA inhibited the formation of peroxides independently of the almond percentage used in emulsion. This inhibition increases with the increase of BHA concentration.

The presence of thyme waste extract strongly influences peroxide formation in all types of emulsions in the same way as BHA. The peroxide levels decreased with the increase in thyme waste extract concentration (Figure 3d), this is due to the same antioxidant effect exerted by this extract. Sinapic, ferulic, and caffeic acids, present in the thyme waste extract are considered good antioxidants, having been some of these hydroxycinnamic acid derivatives used as natural antioxidants in food, beverages, and cosmetics [24]. 

Figure 3e,f depict, respectively, the combined effect of BHA and thyme waste extract concentrations with the time storage on hydroperoxides formation. Thyme waste extract and BHA had the same behavior on peroxide formation during storage time. Hydroperoxides increased during time in all O/W emulsions. As compared to the BHA, thyme waste extract was more or less similarly efficient on controlling the evolution of primary oxidation products for almost all formulations. It is noteworthy that the presence of wheat germ oil at higher concentration mostly affects the emulsion stability being more prone to oxidation due to the predominance of unsaturated fatty acids [3].

### 3.5. Secondary Lipid Oxidation Products in Formulated Emulsions

Lipid and oil based formulations are susceptible to degradation through lipid peroxidation which represents a concerning chemical instability [25]. The aim of this part of the work is to evaluate the antioxidant effectiveness of thyme waste extract on MDA formation during oxidation of wheat and almond oil emulsion. 

**Figure 4:**
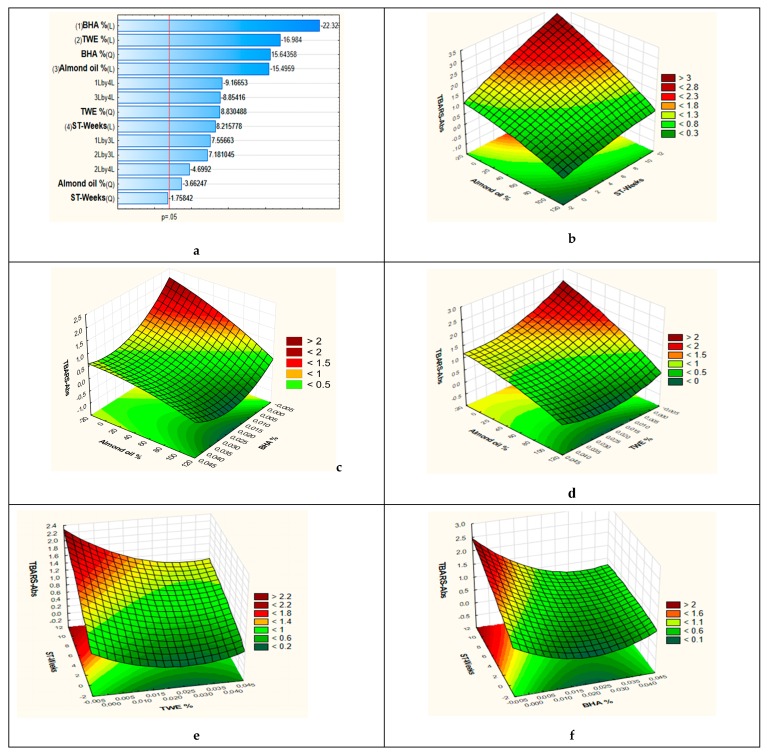
(**a**): Pareto chart showing the effects of the significant model coefficients. (**b**): Response surface plot showing the combined effect of storage time and emulsion almond oil %. (**c**): Response surface plot showing the combined effect of BHA concentration and emulsion almond oil %. (**d**): Response surface plot showing the combined effect of thyme extract concentration and emulsion almond oil %. (**e**): Response surface plot showing the combined effect of thyme extract concentration and storage time.(**f**): Response surface plot showing the combined effect of BHA concentration and storage time. TWE: Thyme waste extract; ST: storage time.

In order to examine the relative importance of the main effects and their interactions with statistical significance (*p* < 0.05), a standardized Pareto chart (Figure 4a) was employed. The results showed that the main factors (1) BHA concentration (L), (2) thyme waste extract concentration (L), (3) Almond oil % (L), (4) Storage time (L) and their interactions [BHA concentration (Q Thyme waste extract concentration (Q), Almond oil % (Q), 1 L by 4 L, 3 L by 4 L, 1 L by 3 L, 2 L by 3 L, 2 L by 4 L) that extend beyond the reference line were significant at the level of 0.05. BHA followed by thyme waste extract concentration presented the most significant factors, which affected oxidative stability of lipids in the investigated emulsion. All other factors and their interactions had less effect but were statistically significant at 95% confidence, except for the interaction of storage time (Q) expressed in weeks.

Initially (Figure 4b), interaction between factors like almond oil % and 10 weeks of storage time were examined to determine their impact on the secondary lipid oxidation expressed as absorbance. As it can be noticed in the plot, there is an increase in the absorbance with the increase of storage time and with the decrease of almond oil proportion in the emulsion. The increase of almond proportion in the emulsion enhances its resistance to oxidation. The lipid oxidation was shown to be low during the entire studied storage time when almond oil proportion in the emulsion was between 75 to 100%, whereas the inverse was observed between 0% to 25%.

The response surfaces obtained for secondary lipid oxidation as a function of the percentage of almond oil and BHA concentration in emulsion are illustrated in Figure 4c. A significant increase of the lipid oxidation was observed along with the decrease of almond oil proportion for all BHA concentrations. This increase was extremely important in the absence of BHA. Figure 4d shows the response surface plot depicting the effect of thyme waste extract concentration and almond oil on secondary lipid oxidation. As seen for BHA concentration in emulsions, lipid oxidation decreases with the increase of thyme waste extracts until optimal concentration of 0.02% to 0.03% is reached, exhibiting the lowest absorbance, after which the phenomenon started to reverse. When compared to the BHA, thyme extract was more or less similarly efficient in controlling the evolution of MDA for almost all formulations. Furthermore, whatever the antioxidant used, values gradually increased during the storage period by raising the percentage of wheat germ oil. 

According to Gallego et al. [26], the emulsion stability increased with increasing concentration of natural extracts. Emulsions containing extract concentrations of 0.02% and 0.2% showed a higher stability than those prepared with the positive Trolox control (0.02%), and the 0.2% extract exhibited a similar antioxidant effect to that of BHA, at 0.004%. Besides, Poyato et al. [27] have shown that the type of antioxidant is a key factor in the control of oxidation process in W/O/W or O/W emulsions, which are formulated with highly polyunsaturated oils. The Banias et al. [28] study showed that thyme extracts have strong antioxidant effects in stabilizing lard. 

Formulations with the combination of higher concentrations of thyme waste extract (0.02%, 0.04%) and almond oil (≥50%) were the best in protecting the oxidation of emulsion samples. According to Yun and Surh [29], the ratio of oleic acid to linoleic acid could be used as a criterion for determining oil stability and fatty acid composition. Oleic acid (C18:1) has been estimated to be 10 to 40 times less susceptible to oxidation than linoleic acid (C18:2). 

The effects of storage time with each of the two antioxidants (BHA and thyme extract) on emulsion lipid oxidation are displayed as 3D surface plot in Figure 4e,f, respectively. As shown, lipid oxidation increases with the storage time in the same way for both antioxidants. This oxidation was slower in the presence of any of the two antioxidants.

## 4. Conclusions

Thyme waste extract prevented the hydroperoxide formation and the accumulation of malondialdehyde in emulsions O/W, nevertheless this was dependent on its concentration as well as on the ratio of wheat germ and almond oil at the oil phase. The combination of higher concentrations of thyme waste extract (0.02%, 0.04%) and almond oil (≥50%) were the best in protecting the primary oxidation of emulsion samples (prevention of hydroperoxide formation). Thyme waste extract was only effective in the prevention of the accumulation of malondialdehyde when 100% almond oil was used; the presence of wheat germ hampered the antioxidant action of the thyme waste extract. Over the storage period (10 weeks), the antioxidant ability of thyme waste extract decreased, becoming more accentuated as the concentration of wheat germ oil increased. Depending on the ratio of oils (wheat germ and almond oil), there was also a change in the viscosity trend of emulsions O/W as the antioxidant was added: for all the thyme waste extract concentrations, the initial viscosity of the samples increased with the decrease of wheat germ oil percentage, from 100% to 50%; after this ratio, the initial viscosity decreased by increasing the almond oil percentage. The results indicate that the extract of waste thyme obtained after steam distillation for producing essential oils can constitute a good antioxidant for stabilizing diverse types of materials such as emulsions O/W, provided that the oil phase is not predominantly constituted by polyunsaturated fats.

## Figures and Tables

**Table 1:** Chemical composition of thyme waste extract. Total polyphenols content and DPPH activity of thyme waste extract.

No.	Retention Time RT (time)	Relative Area Percent (%)	Compounds	*m*/*z* (I)
**1**	7.05	40.88	Benzoic acid	105(100); 179(93); 77(93); 135(82); 51(22); 180(13); 136(11); 73(8); 106(7); 75(7)
**2**	10.93	11.89	*trans*-Cinnamic acid (TMS)	131(100); 205(95); 103(95); 161(85); 77(74); 75(75); 145(45); 102(43); 135(25); 73(24)
**3**	11.83	17.18	4-Hydroxybenzoic acid (2TMS)	73(100); 267(72); 223(68); 193(46); 268(16); 224(15); 282(14); 75(12); 126(10); 91(10)
**4**	15.16	10.34	*p*-Coumaric acid (TMS)	73(100); 293(43); 219(40); 75(30); 249(30); 308(24); 179(11); 294 (10); 74(10); 220(8)
**5**	16.40	2.55	Ferulic acid (2TMS)	73(100); 338(45); 323(20); 75(34); 308(34); 249(30); 293(30); 59 (13); 219(13); 339(12)
**6**	16.70	8.74	Caffeic acid (TMS)	73(100); 219(68); 396(38); 191(16); 381(13); 397 (13); 220(12); 75(11); 74(8); 249(7)
**7**	17.37	3.71	Sinapic acid (2TMS)	73(100); 338(57); 368(45); 75(34); 353(32); 232(25); 279(15); 339(15); 59(14); 249(12)
**8**	21.91	0.90	Quercetin (5TMS)	73(100); 647(29); 648(16); 649(9); 559(8); 74(7); 75(6); 560(3); 147(3); 575(2)
Total *		96.19		
* Only the most abundant peaks were identified.				
I: intensity				
**Phenols (mg EAG/g)**		**Flavones (mg EQ/g)**	**Dihydroflavonols (mg EE/g)**	**DPPH (IC_50_ mg/mL)**
97.50 ± 3.82		0.13 ± 0.01	1.76 ± 0.24	0.093 ± 0.01

## Supplementary Materials

The following are available online at https://www.mdpi.com/2076-3921/8/8/243/s1, Table S1. Apparent viscosity of the emulsions with thyme extract and BHA, at different concentrations (0.01, 0.02 and 0.04%), under storage conditions of 10 weeks at 37 °C. Apparent viscosity (mPa.s) was determined at a shear rate of 6.3 s^−1^, with a LV-3C spindle. Values of mean (*n* = 4) ± standard deviation.
