# Supplementary material for: Antioxidant Activity of Thyme Waste Extract in O/W Emulsions
Source: Antioxidants (Basel). 2019 Jul 25;8(8):243. doi: 10.3390/antiox8080243 (PMC6719112; doi:10.3390/antiox8080243)
Supplement: Supplementary file 1 [file antioxidants-08-00243-s001.pdf]

Antioxidant activity of thyme waste extract in O/W emulsions

Soukaina El-Guendouz[1,2], Smail Aazza[3], Susana Anahi Dandlen[2], Nessrine Majdoub[2], Badiaa Lyoussi[1] , Sara Raposo[4], Maria Dulce Antunes[5], Vera Gomes[6], Maria Graça Miguel[2*]

[1]Laboratory of Physiology-Pharmacology-Environmental Health, Faculty of Sciences Dhar El Mehraz, BP 1796 Atlas, University Sidi Mohamed Ben Abdallah, Fez 30 000, Morocco.
[2]Department of Chemistry and Pharmacy, MeditBio, Faculty of Science and Technology, University of Algarve, Campus de Gambelas, 8005-139 Faro, Portugal.
[3]Laboratory of phytochemistry, National Agency of Medicinal and Aromatic Plants (ANPMA). BP. 159, Principal, 34000, Taounate. Morocco.
[4]Centre for Marine and Environmental Research (CIMA), Faculty of Sciences and Technology, University of Algarve, Campus de Gambelas, 8005-139 Faro. Portugal.
[5]MeditBio / CEOT, Faculty of Science and Technology, University of Algarve, Campus de Gambelas, 8005-139 Faro, Portugal.
[6]Centre of Marine Sciences (CCMAR), University of Algarve, Campus de Gambelas, 8005-139 Faro, Portugal

Correspondence: mgmiguel@ualg.pt

Table S1. Apparent viscosity of the emulsions with thyme extract and BHA, at different concentrations (0.01, 0.02 and 0.04 %), under storage conditions of 10 weeks at 37 °C. Apparent viscosity (mPa.s) was determined at a shear rate of 6.3 s^{-1}, with a LV-3C spindle. Values of mean (n=4) ± standard deviation.

		**A 100% of Germ wheat oil / 0% almond oil**		**B 75% of Germ wheat oil / 25% almond oil**		**C 50% of Germ wheat oil / 50% almond oil**		**D 25% of Germ wheat oil / 75% almond oil**		**E 0% of Germ wheat oil / 100% almond oil**	
		**Week 0**	**Week 10**	**Week 0**	**Week 10**	**Week 0**	**Week 10**	**Week 0**	**Week 10**	**Week 0**	**Week 10**
0.01 %	Control	2020±34	1834±59	1852±23	1884±57	1796±06	1872±57	1844±28	1776±51	1826±37	1736±34
	Thymus	1823±10	1744±15	1984±24	1776±37	2028±21	1780±39	1920±18	1696±27	1904±41	1720±27
	BHA	1760±00	1936±34	1822±14	2004±85	1754±25	1844±45	1820±34	1858±65	1784±34	1832±45
0.02 %	Control	2020±34	1834±59	1852±23	1884±57	1796±06	1872±57	1844±28	1776±51	1826±37	1736±34
	Thymus	1720±42	2132±11	1723±15	2058±69	1860±14	2116±87	1848±57	2136±24	1848±34	1988±34
	BHA	1640±51	1716±85	1716±23	1722±37	1684±57	1676±28	1634±14	1642±31	1594±08	1620±23
0.04 %	Control	2020±34	1834±59	1852±23	1884±57	1796±06	1872±57	1844±28	1776±51	1826±37	1736±34
	Thymus	1884±35	2256±47	2060±84	2399±34	2080±97	2451±37	1824±54	2144±49	2008±23	2156±66
	BHA	1680±68	2046±42	1418±42	1708±62	1558±54	1700±40	1526±65	1685±37	1546±31	1692±74
